# Incidence of phlebitis associated with the use of peripheral IV catheter and following catheter removal

**DOI:** 10.1590/1518-8345.0604.2746

**Published:** 2016-08-08

**Authors:** Janete de Souza Urbanetto, Cibelle Grassmann Peixoto, Tássia Amanda May

**Affiliations:** 1 PhD, Adjunct Professor, Faculdade de Enfermagem, Nutrição e Fisioterapia, Pontifícia Universidade Católica do Rio Grande do Sul, Porto Alegre, RS, Brazil.; 2 RN, Hospital São Lucas, Pontifícia Universidade Católica do Rio Grande do Sul, Porto Alegre, RS, Brazil.; 3 RN.

**Keywords:** Flebitis, Seguridad del Paciente, Enfermería, Infusiones Intravenosas

## Abstract

**Objective:**

to investigate the incidence of phlebitis and its association with risk factors when using peripheral IV catheters (PIC) and following their removal - (post-infusion phlebitis) in hospitalized adults.

**Method:**

a cohort study of 171 patients using PIC, totaling 361 punctures. Sociodemographic variables and variables associated with the catheter were collected. Descriptive and analytical statistical analyses were performed.

**Results:**

average patient age was 56.96 and 51.5% of the sample population was male. The incidence of phlebitis was 1.25% while using PIC, and 1.38% post-infusion. The incidence of phlebitis while using PIC was associated with the length of time the catheter remained in place, whereas post-infusion phlebitis was associated with puncture in the forearm. Ceftriaxone, Clarithromycin and Oxacillin are associated with post-infusion phlebitis.

**Conclusions:**

this study made it possible to investigate the association between risk factors and phlebitis during catheter use and following its removal. The frequency of post-infusion phlebitis was larger than the incidence of phlebitis with the catheter in place, with Phlebitis Grade III and II being the most frequently found in each of these situations, respectively. Aspects related to post-infusion phlebitis can be explained, given the limited number of studies addressing this theme from this perspective.

## Introduction

Peripheral IV catheterization (PIC) is the most common invasive procedure performed on hospitalized patients^1^. It requires manual dexterity and tec hnical competence, knowledge of pharmaceutical therapy and familiarity with the anatomy and physiology of the vascular system. Because catheterization is done for different purposes and for different lengths of time, it represents a potential risk for a number of safety incidents, including microbial growth^2^. However, regardless of the generating factor, local complications take the form of bruises, infiltration, leakage, catheter obstruction and phlebitis^3^.

Phlebitis is an inflammation of the vein, which may bring with it pain, erythema, edema, hardening and/or a palpable thread^4^. Numerous factors can influence the development of phlebitis, such as inadequate technique when inserting the catheter, the patient's clinical situation, the characteristics of the vein, drug incompatibility, tonus and pH of the medicine or solution, ineffective filtration, catheter diameter, size, length and material of manufacture; prolonged use^3^ ^,^ ^5^ ^-^ ^6^.

Phlebitis can be split into four types ^7^ ^-^ ^8^: mechanical, when movement of the cannula inside the vein causes friction and inflammation, or when the cannula is too wide for the vein; chemical phlebitis, caused by the drug or fluid infused through the catheter, where factors such as pH and osmolality can significantly impact the incidence of phlebitis; bacterial, when bacteria penetrates the vein, starting as an inflammatory response to catheter insertion and subsequent colonization of the site by bacteria. Bacterial phlebitis can create serious complications due to the potential for the development of systemic sepsis^7^. Post-infusion phlebitis normally appears 48 to 96 hours after the catheter is removed. Incidence is related especially to catheter material and the length of time the catheter remained in the patient's vein^8^.

Phlebitis manifests in four grades: Grade 1 - erythema around the puncture site, with or without local pain; Grade 2 - pain at the puncture site with erythema and/or edema and hardening; Grade 3: pain at the puncture site with erythema, hardening and a palpable venous cord; Grade 4: pain at the puncture site with erythema, hardening and a palpable venous cord that is > 1 cm, with purulent discharge.

A search of the LILACS and SCIELO databases between 2003 and 2014 using "phlebitis" as the search criterion found 16 and 18 articles respectively, of which four and five respectively were relevant to this analysis, including repetitions. Only one of these had post-infusion phlebitis as a topic.

The incidence of phlebitis in the literature varies quite a bit, with reports ranging from 61.2%^9^ to 1.3^%(^ ^10^. The acceptable rate in any given population of patients is at most 5%^11^. Thus, this study is justified due to the need to monitor and track the incidence of phlebitis in this teaching institution.

By analyzing the aspects above, we found gaps in the knowledge of the incidence of phlebitis, especially post-infusion phlebitis. Given the need for research on this topic and its importance as an indicator of the quality of nursing care, the goal of this study is to investigate the incidence of phlebitis and the association between risk factors and the incidence of phlebitis while using and following the removal of PIC (post-infusion phlebitis) in hospitalized adults.

## Method

This is a cohort study. The study population is made up of 171 adult patients (aged 18 or over) hospitalized in a clinical hospitalization service of a university hospital in the city of Porto Alegre. Inclusion criteria were the use of peripheral intravenous catheter during hospitalization, assessment of the catheter in the first 12 hours following insertion, and consent to participate in the study. Data was collected in October and November 2013. The total patients in the sample allowed us to analyze 361 punctures for the placement of peripheral IV catheters. 

Data was collected by researchers using a tool with the following variables: sociodemographic data (age and gender), and data related to the PIC (date of puncture, location of insertion, catheter gauge (G), permanence (hours), and IV medication being administered. The location where the catheter was inserted was examined daily for the signs and symptoms of phlebitis. The location was monitored for up to 96 hours following catheter removal. Each PIC was analyzed individually as a new case. Phlebitis was categorized based on the moment symptoms appeared - before or after catheter removal, in which case it is known as post-infusion. 

The drugs followed during this study were those stated as being related to phlebitis^12^: antibiotics (Clavulanic acid + Amoxicillin, Ampicillin, Amphotericin B, Aztreonam, Cephalotin, Cefazolin, Cefepime, Cefotaxime, Cefoxitin, Ceftazidime, Ceftriaxone, Cefuroxime, Clarithromycin, Erythromycin, Ertapenem, Imipinem, Levofloxacin, Meropenem, Oxacillin, Piperacillin + Tazobactam, Sulfamethoxazole + Trimethoprim, Ticarcillin + Clavulanic Acid, Tigecycline, Vancomycin); anti-virals (Acyclovir, Ganciclovir); anti-arrhythmics (Amiodarone); anti-spasmodics (Dantrolene); hypnotic sedatives (Diazepam, Promethazine); vasoconstrictors (Dobutamine); vasolidators (Nitroglycerin); anti-epileptics (Phenytoin, Phenobarbital); narcotic analgesics (Fentanyl, Meperidine); fat soluble vitamins (Phytomenadione); anti-anemics (Ferric Hydroxide); sedatives (Midazolam); antacids (Pantoprazole) and anti-mycotics (Voriconazole).

For a descriptive analysis of the data we used central tendency and dispersion measurements (mean and standard deviation) and proportions (percentages). For inferential analyses we used association tests (Chi-squared and Fischer). We adopted a significance level of p<0.05. We used SPSS (Statistical Package for Social Sciences, SPSS Inc, Chicago), Windows version 17.0 for statistical data analysis. 

To calculate the incidence of phlebitis we divided the number of cases over the period by the number of patients/day with peripheral venous access in the same period, multiplied by 100^13^. The average number of patients with PIC each day was 48, totaling 2,880 patients over the 60-day period (two months). Incidence was calculated for phlebitis in general and phlebitis with catheter in place and following catheter removal. 

This Project was approved by the institution's Research Ethics Committee (Protocol 07/03893). All of the patients signed the Free and Informed Consent Form. Whenever signs of phlebitis were identified, the nurse in charge was informed and measures implemented as per the unit's SOP.

## Results

The results of this study enabled a number of analyses that may contributed to understanding this complication resulting from the use of IV catheters. Most of the patients in the study were male, with an average age of 56.96 ±18.46, and median of 58 (18 - 98). Of the 361 catheters assessed, the average time they remained in place was 3.37 ± 1.11 days, and the median 3 days (1 - 6). The average number of catheters per patient was 2.1 ± 1.62; 53.2% (n = 91) of the patients used a single catheter; 19.9% (n = 34) used two, 11.1% (n = 19) three; and 13.5% (n = 27) between four and eight catheters. There was no relationship between the number of catheters used and the incidence of or grade of phlebitis (p=0.572 and p=0.974 respectively), or following catheter removal (p=0.120 and p=0.569 respectively). Table 1 shows the descriptive data of age and gender, and those related to the use of PICs.

**Table 1:** Characteristics of age and gender related to PIC, location of puncture and drugs being used. Porto Alegre/RS, Brazil, 2014. n = 171 patients.

Characteristics		N	%
Age			
	≤ 57	83	48.5
	≥ 58	88	51.5
Gender			
	Female	83	48.5
	Male	88	51.5
Type of catheter (n=361)			
	Catheter with mandrel	361	100
Catheter gauge (n=361)			
	18 gauge	01	3.0
	20 gauge	19	5.3
	22 gauge	183	50.7
	24 gauge	158	43.8
Catheter Maintenance (n=361)			
	Saline/Intermittent	279	77.3
	Continuous saline	82	22.7
Time catheter remained in place (n=361)			
	Less than 72 hours	194	53.7
	Over 72 hours	167	46.3
Location of Catheter Puncture (n=361)			
	Forearm	7	1.9
	Elbow Pit	63	17.5
	Arm/Writs	200	55.4
	Hand	91	25.2
Drugs (n=361)			
	Antiviral	30	8.3
	Antiarrhythmic	10	2.8
	Vasoconstrictor	01	0.3
	Antiepileptic	36	10.0
	Antacid	06	1.7
	Anti-anemic	20	5.5
	Vitamin	18	5.0
	Sedative/Analgesic	210	58.2
	Antibiotic	184	51,0

Source: Study data

Table 2 shows the frequency and trade of phlebitis during use and following removal of the PICs. The total incidence of phlebitis during use and following removal of the PICs was obtained by dividing the total number of phlebitis cases (76) by the total number of patients/day using PIC over the period (2,880) and multiplying by 100. The result was 2.63%. Using the formula above, the incidence of phlebitis while using PICs (36) was 1.25%, the incidence of post-infusion phlebitis (following catheter removal) (40) was 1.38%.

Table 3 shows the frequency of phlebitis while using PIC and following PIC removal, and the association of the incidence and grade of phlebitis with the risk factors monitored in this study. The length of time the catheter remained in place (≥ 72 hours) was associated with the incidence of phlebitis (p = 0.016), and puncture in the forearm was associated with post-infusion phlebitis (p = 0.054). Other factors, including grade, did not demonstrate any association with the incidence of the inflammation.

**Table 2:** Descriptive analysis of phlebitis. Incidence, type and grade. Porto Alegre/RS, Brazil, 2014. n = 171 patients.

		N	%
Signs of phlebitis while using PIC* (n=361)			
	Yes	36	10.0
	No	325	90.0
Phlebitis grade while using PIC* (n=36)			
	Grade I	25	69.4
	Grade II	09	25.0
	Grade III	02	5.5
	Grade IV	-	-
Post-infusion (following PIC removal*) phlebitis (n=361)			
	Yes	40	11.1
	No	321	88.9
Phlebitis grade following PIC* removal (n=40)			
	Grade I	17	42.5
	Grade II	22	55.0
	Grade III	01	2.5
	Grade IV	-	-

PIC* - Peripheral IV Catheter

**Table 3:**
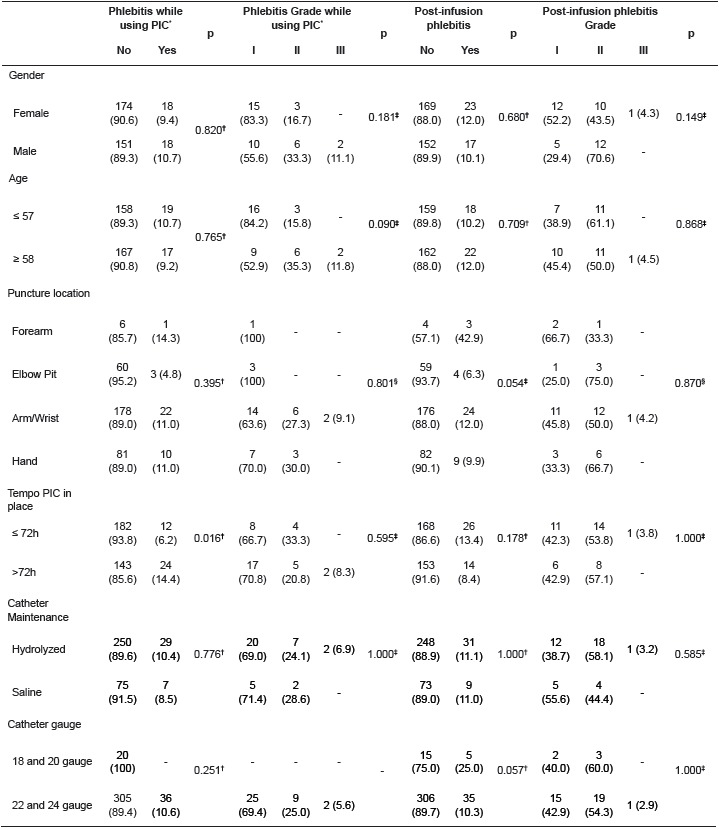
Frequency and association between age, gender and PIC insertion characteristics and the incidence of phlebitis during PIC use and following its catheter removal. Porto Alegre/RS, Brazil, 2014. n= 361 peripheral venous accesses.

PIC* - Peripheral IV Catheter † Chi-squared with correction for continuity; ‡Fisher Test; § Chi Squared test. Source: Study data

Table 4 shows the results of the association of phlebitis during catheter use and following withdrawal and the use of drugs, by group. None of the groups of drugs studied showed any association with phlebitis. However, when we looked at specific drugs within each group, Ceftriaxone (n=7; 25%), Clarithromycin (n=7; 28%) and Oxacillin (n=6; 46.2%) were associated with post-infusion phlebitis (p = 0.033; p = 0.014 and p ≤ 0.001 respectively).

**Table 4:** Frequency and association of drugs, by drug group, with the incidence of phlebitis while using and following removal of PICs. Porto Alegre/RS, Brazil, 2014. n= 361 peripheral venous accesses.

		Phlebitis while using PIC^*^		P	Phlebitis Grade while using PIC^*^			P	Post-infusion phlebitis		p	Post-infusion phlebitis Grade			P
		No	Yes		I	II	III		No	Yes		I	II	III	
Antiviral															
	No	287 (89.7)	33 (10.3)	0.745^†^	23 (69.7)	8 (24.2)	2 (6.1)	1.000^‡^	281 (87.8)	39 (12.2)	0.108^†^	16 (41.0)	22 (56.4)	1 (2.6)	0.451^‡^
	Yes	38 (92.7)	3 (7.3)		2 (66.7)	1 (33.3)	-		40 (97.6)	1 (2.4)		1 (100)	-	-	
Antiarrhythmic															
	No	317 (90.3)	34 (9.7)	0.590^†^	25 (73.5)	7 (20.6)	2 (5.9)	0.091^‡^	313 (89.2)	38 (10.8)	0.689^†^	16 (42.1)	21 (55.3)	1 (2.6)	1.000^‡^
	Yes	8 (80.0)	2 (20.0)		-	2 (100)	-		8 (80.0)	2 (20.0)		1 (50.0)	1 (50.0)	-	
Vasoconstrictor															
	No	325 (90.3)	35 (9.7)	0.181^†^	24 (68.6)	9 (25.7)	2 (5.7)	1.000^‡^	320 (88.9)	40 (11.1)	1.000^†^	17 (42.5)	22 (55.0)	1 (2.5)	-
	Yes	-	1 (100)		1 (100)	-	-		1 (100)	-		-	-	-	
Anti-epileptic															
	No	294 (90.5)	31 (9.5)	0.594^†^	20 (64.5)	9 (29)	2 (6.5)	0.487^‡^	287 (88.3)	38 (11.7)	0.405^†^	16 (42.1)	21 (55.3)	1 (2.6)	1.000^‡^
	Yes	31 (86.1)	5 (13.9)		5 (100)	-	-		34 (94.4)	2 (5.6)		1 (50.0)	1 (50.0)	-	
Antacids															
	No	319 (89.9)	36 (10.1)	0.893^†^	25 (69.4)	9 (25.0)	2 (5.6)	-	315 (88.7)	40 (11.3)	0.829^†^	17 (42.5)	22 (55.0)	1 (2.5)	-
	Yes	6(100)	-		-	-	-		6(100)	-		-	-	-	
Anti-anemic															
	No	309 (90.9)	31 (9.1)	0.248^†^	22 (71.0)	8 (25.8)	1 (3.2)	1.000^‡^	302 (88.8)	38 (11.2)	1.000^†^	17 (44.7)	20 (52.6)	1 (2.6)	0.520^‡^
	Sim	16 (76.2)	5 (23.8)		3 (60.0)	1 (20.0)	1 (20.0)		18 (90.0)	2 (10.0)		-	2(100)	-	
Vitamins															
	No	309 (90.1)	34 (9.9)	1.000^†^	23 (67.6)	9 (26.5)	2 (5.9)	1.000^‡^	304 (88.6)	39 (11.4)	0.703^†^	17 (43.6)	21 (53.8)	1 (2.6)	1.000^‡^
	Yes	16 (88.9)	2 (11.1)		2 (100)	-	-		17 (94.4)	1 (5.6)		-	1 (100)	-	
Sedative/analgesic															
	No	135 (89.4)	16 (10.6)	0.875^†^	8 (50.0)	6 (37.5)	2 (12.5)	0.062^‡^	137 (90.7)	14 (9.3)	0.448^†^	5 (35.7)	9 (64.3)	-	0.692^‡^
	Yes	190 (90.5)	20 (9.5)		17 (85.0)	3 (15.0)	-		184 (87.6)	26 (12.4)		12 (46.2)	13 (50.0)	1 (3.8)	
Antibiotics															
	No	163 (93.1)	12 (6.9)	0.132^§^	7 (58.3)	4 (33.3)	1 (8.3)	0.486^‡^	160 (91.4)	15 (8.6)	0.281^§^	8 (53.3)	6 (40.0)	1 (6.7)	0.237^‡^
	Yes	160 (87.0)	24 (13.0)		18 (75.0)	5 (20.8)	1 (4.2)		159 (86.4)	25 (13.6)		9 (36.0)	16 (64.0)	-	

PIC* - Peripheral IV Catheter † Chi-squared with correction for continuity; ‡Fisher Test; § Chi Squared test. Source: Study data

## Discussion

The results of following 171 hospitalized patients using PIC enabled important analyses that can contribute to elucidating a number of aspects related to the incidence of phlebitis during intravenous therapy.

Regarding incidence, our study showed that total incidence (2.63%) and the incidence of phlebitis during PIC use (1.25%) and following catheter removal (1.38%) were within the international guidelines of the Intravenous Nurse Society^4^, or less than 5%. Compared to other studies^(10,14)^, we found a wide variation in incidence, from 1.3% to 25.8%. This may be due to the different methods used and the specific limitations of each study.

We found a higher incidence of phlebitis after catheter removal (1.38%) than when the PIC was in place. Literature searches found no data comparing the incidence of phlebitis during PIC use and after removal, which demonstrates the need for further studies on this topic, and the importance of monitoring the insertion site following catheter removal, a procedure that is not well disseminated and that makes a major difference in early identification of post-infusion phlebitis. This etiology is likely due to an inflammatory reaction starting close to the moment when the catheter was removed, with still with no visible symptoms. This should be considered in protocols so as not to underestimate the incidence or prevalence of phlebitis in the institution. 

Regarding Grade, the most frequent grade of phlebitis found during catheter use was Grade II, while Grade III was the most common in post-infusion phlebitis. Other studies corroborate these findings, with Grade I and II phlebitis being more common with PICs in place^3^ ^,^ ^6^. We found no studies elucidating the Grade of phlebitis in post-infusion phlebitis, once again showing the need to investigate this topic and train nursing teams in the specificities of post-infusion phlebitis.

Of the 361 PIC punctures analyzed, the average permanence of the catheters was 3.37 ± 1.11 days, and the median 3 days, as recommended by ANVISA and the Royal College of Nursing^,(^ ^15^ ^-^ ^16^. Of the 167 catheters remaining for more than 72 hours, 24 patients (14.4%) showed the signs and symptoms of phlebitis. There was a significant (p=0.016) incidence of phlebitis compared to those who did not develop phlebitis. We found that the length of time the catheter remains in place influences the appearance of phlebitis, as found in another study, where the incidence of phlebitis was 62.5% when PICs remained in place longer than 72 hours.

Regarding gender and age, we found no statistical association with the incidence of phlebitis, unlike another study^8^, claiming that one of the risk factors is being older than 65. However, in terms of gender it agrees with the study^9^ that states there is no association between gender and phlebitis. 

Although the forearm is the preferred location for puncture due to its thick veins^6^, only 1.9% of the punctures in this study were in the forearm. On the other hand, this location was of limiting significance (p=0.054) in terms of developing phlebitis, when compared to other locations. Another study found no significant association between part of the body and phlebitis^3^, however the forearm was the location of puncture most often used by the nursing team.

When we analyzed the incidence of phlebitis against the gauge of the IV catheter, we found the most frequently used gauges were 22G and 24G (94.5%). We found limiting significance (p=0.057) in the incidence of post-infusion phlebitis when larger caliber catheters were used (18G and 20G). These findings coincide with those of another study^3^, where 65% of the phlebitis cases were in patients in which 18G and 20G catheters had been used, unlike a previous study ^17^ that found a higher incidence of phlebitis (80.7%) when using 22G and 24G catheters. 

When we looked at the therapeutic class of the drugs we monitored, 51.0% of the patients used antibiotics while the PIC was in place, however we found no significant association with the incidence of phlebitis. Yet when looking at each drug individually, Ceftriaxone (p=0.033), Clarithromycin (p=0.014) and Oxacillin (p≤ 0.001) showed an association with post-infusion phlebitis. Looking at the package leaflets for these drugs^18^, we found that phlebitis was listed as a possible adverse reaction only for Ceftriaxone and Oxacillin, which is in agreement with the findings of the present study. 

Other medicines, while not showing any significant association, did have relevant results, such as amiodarone and ferric hydroxide, where we found a higher percentage of the signs of phlebitis in patients using these medicines (20.0% and 23.8% respectively) than in those who did not. We also found a higher percentage of post-infusion phlebitis among patients who used vancomycin (33.3%) than among not making use of this medication. In terms of the pH of the medicines, the more acidic the higher the risk of chemical phlebitis^19^, which is in agreement with the findings regarding oxacillin (pH of 4.5 - 7.5)^20^, however this does not explain the findings with other drugs. 

This study shows the importance of continued follow-up of the insertion site, as post-infusion phlebitis is not normally monitored by the institutions, and thus not computed in the incidence or prevalence rates used. Monitoring the insertion site (following catheter removal) is also important as phlebitis may result in longer hospitalization times as it is considered a clinical complication, and lead to a higher financial and psychological burden as a result of longer time spent in the hospital.

## Conclusion

This study allowed us to look at the association between risk factors and the incidence of phlebitis during the use and following the removal of peripheral IV catheters, showing the frequency of post-infusion phlebitis was larger than the incidence of phlebitis with the catheter in place, and that Grades III and II were the most frequent, respectively. Based on this finding, we infer there is a need to systematically monitor indicators in our searched for continued quality of care, and to compare rates in the different contexts of nursing practice. 

It is possible that a reduction in osmolarity resulting from the correct dilution when administering these drugs, as this institution follows the recommendations in the pharmacotherapeutic handbook, may have contributed to not finding a greater association between the drugs investigated and phlebitis. However, this aspect was not controlled in this study. We also found it difficult to analyze the insertion site when non-transparent adhesives were used to secure the catheter, or when non-allergenic tape was placed over the PIC site. This may have contributed to the evolution of higher Grades of phlebitis, showing the need for educational strategies to teach the staff how to properly use catheter, and to hold them in place with suitable material. 

Because the study patients were discharged from the hospital, we lost the ability to track the insertion sites following PIC removal, making it impossible to follow some of the patients for 96 hours, which may have contributed to a smaller number of post-infusion phlebitis cases.

This study helped elucidate aspects related to the incidence of post-infusion phlebitis. It is important to intensify education and training in early identification of phlebitis, and to monitor the insertion site after the catheter is removed, as few studies have addressed this topic from this perspective.
